# Profilaktyczna Obustronna Salpingektomia: Dlaczego Warto?

**DOI:** 10.34763/devperiodmed.20182204.390395

**Published:** 2019-01-14

**Authors:** Łukasz Kurek, Krzysztof Jabłoński, Natalia Góralska, Joanna Żuchlińska, Łukasz Stelmaszczyk, Paweł Różanowski

**Affiliations:** 1 Katedra Anatomii, Collegium Medicum, Uniwersytet Warmińsko-Mazurski w Olsztynie, Olsztynie, Polska; 2 Klinika Ginekologii i Położnictwa, Collegium Medicum, Uniwersytet Warmińsko-Mazurski w Olsztynie, Olsztynie, Polska; 3 Oddział Kliniczny Onkologii i Immunoonkologii, Warmińsko-Mazurskie Centrum Onkologii, Szpital Ministerstwa Spraw Wewnętrznych i Administracji w Olsztynie, Olsztynie, Polska

**Keywords:** rak jajnika, jajowody, prewencja, ovarian cancer, fallopian tubes, prevention

## Abstract

Nowotwory złośliwe jajnika charakteryzują się późną wykrywalnością, co jest głównym czynnikiem determinującym ich złe rokowanie. Prawie 70% z nich rozpoznawanych jest w III i IV stadium zaawansowania klinicznego. Obecnie brakuje skutecznych metod wczesnego wykrywania raka jajnika. Jednym ze sposobów obniżenia ryzyka zachorowania jest stosowanie obustronnego usunięcia jajowodów i jajników, a coraz częściej proponuje się, że skutecznym postępowaniem może być profilaktyczna salpingektomia. Jest to uzasadnione badaniami patomorfologicznymi dotyczącymi pochodzenia komórek nowotworowych w raku jajnika. Istnieją dowody naukowe sugerujące, że często pierwotnym miejscem wystąpienia przemian nowotworowych jest dystalny odcinek jajowodu, z którego komórki następnie migrują do jajników. Brakuje jednak długoterminowych prac dowodzących skuteczności profilaktycznej salpingektomii. Poniższy artkuł podsumowuje korzyści i wady takiego postępowania na podstawie analizy aktualnego piśmiennictwa.

## Abstract (en)

Malignant tumors of the ovary are characterized by late detection which is the chief factor responsible for their poor prognosis. Almost 70% of ovarian cancers are diagnosed at clinical stage III or IV. At present, effective methods for early detection of ovarian cancer are lacking. One accepted approach to reduce the risk of ovarian cancer is bilateral salpingo-oophorectomy. However, it is increasingly widely believed that even a more limited surgery of bilateral salpingectomy may be also used prophylactically as an effective means to reduce cancer risk. The procedure is based on extensive anatomopathological research on the origins of malignant cells in ovarian cancer. There is already ample scientific evidence that in a high proportion of cases the primary site of neoplastic transformation is the distal segment of the fallopian tube, from which malignant cells migrate to the ovary. However, long-term studies demonstrating the effectiveness of prophylactic salpingectomy for ovarian cancer are required. This article summarizes the benefits and disadvantages of the procedure based on currently available literature

## Wprowadzenie

Nowotwory złośliwe jajnika charakteryzują się późną wykrywalnością co, poza biologią większości nowotworów o tej lokalizacji, jest głównym czynnikiem wpływającym na ich złe rokowanie. W ciągu ostatnich czterech dekad zachorowalność na nowotwory złośliwe jajnika wzrosła u Polek dwukrotnie [1]. Według ostatniego raportu Krajowego Rejestru Nowotworów aktualnie rak jajnika stanowi 4,6% zachorowań (co w liczbach bezwzględnych oznaczało 3735 nowych przypadków w roku 2015) i odpowiada za 6,2% zgonów (odpowiednio – 2768 przypadków) wśród wszystkich nowotworów złośliwych. Po wyłączeniu nowotworów złośliwych skóry, raki jajnika zajmują u Polek piąte miejsce pod względem zapadalności (po nowotworach piersi, płuca, trzonu macicy i jelita grubego) i są czwartą przyczyną zgonów (po nowotworach płuca, piersi i jelita grubego). Większość przypadków schorzenia (60-70%) rozpoznawanych jest w III i IV stopniu zaawansowania klinicznego, dlatego odsetek przeżyć pięcioletnich wśród wszystkich Polek z rozpoznanym nowotworem złośliwym jajnika wynosi jedynie ok. 40% i jest najniższy spośród wszystkich nowotworów ginekologicznych [1]. Brak skutecznych programów przesiewowych uniemożliwia rozpoznanie nowotworów jajnika we wczesnych stadiach zaawansowania, w których odsetki wieloletnich przeżyć sięgają nawet 90%. Na nowotwory złośliwe jajnika chorują przede wszystkim kobiety, które osiągnęły wiek około- i pomenopauzalny, gdyż 80% zachorowań ma miejsce po 50. roku życia. Zapadalność wzrasta z wiekiem, osiągając szczyt w przedziale 55-59 lat. Warto jednak pamiętać, że nowotwory jajnika stanowią 4% zachorowań na nowotwory złośliwe u dzieci i młodzieży do 18 r.ż. oraz ponad 6% zachorowań u tzw. młodych dorosłych kobiet (tj. w wieku 20-44 lat) [1]. Zachorowalność we wcześniejszym wieku dotyczy przede wszystkim kobiet będących nosicielkami mutacji predysponujących. Do uznanych genetycznych czynników ryzyka nowotworów złośliwych jajnika należą: zespoły dziedzicznego raka jajnika i piersi – głównie nosicielstwo mutacji BRCA1 i BRCA2, zespół dziedzicznego raka jajnika, dziedziczny rak jelita grubego niezwiązany z polipowatością (zespół Lyncha II), ponadto bezdzietność lub rzadkie ciąże, długotrwałe stymulacje owulacji, endometrioza, radioterapia obszarów obejmujących miednicę mniejszą, przebycie zakażenia wirusem nagminnego zapalenia przyusznic, stosowanie kosmetyków do higieny intymnej zawierających talk, jak i otyłość [2, 3]. W związku z wysoką śmiertelnością oraz brakiem skutecznych metod screening-u pilnie poszukiwane są sposoby zmniejszenia liczby zachorowań oraz umieralności na nowotwory złośliwe jajnika. Do zdobywających coraz szersze uznanie metod obniżenia ryzyka zachorowania na nowotwór złośliwy jajnika, a w konsekwencji obniżenia śmiertelności z powodu tego nowotworu należą obustronne usunięcie jajników i jajowodów oraz doustna antykoncepcja hormonalna, choć rola tej ostatniej w grupie kobiet nosicielek mutacji BRCA1/2 nie jest jednoznacznie ustalona. Dlatego zdaniem coraz większej grupy autorów jednym z możliwych sposobów ograniczenia zachorowalności na raka jajnika, przynajmniej w wybranych grupach kobiet, może być profilaktyczna obustronna salpingektomia [4]. U źródeł propozycji takiego postępowania leżą coraz liczniejsze dowody na pochodzenie istotnego odsetka nowotworów jajnika właśnie z jajowodów.

Dlatego celem niniejszej pracy jest dokonanie krótkiego przeglądu danych odnośnie zasadności procedury obustronnej salpingektomii (ze szczególnym uwzględnieniem możliwych korzyści i wad) w prewencji raka jajnika, na podstawie analizy aktualnego piśmiennictwa.

## Pochodzenie nowotworów złośliwych jajnika

Zgodnie z podziałem WHO nowotwory złośliwe jajnika dzieli się na nowotwory pochodzenia nabłonkowego (stanowiące 90-95% wszystkich nowotworów złośliwych tego narządu) oraz nienabłonkowe, które z kolei dzieli się na nowotwory pochodzenia germinalnego (to znaczy rozwijające się z pierwotnych komórek płciowych) oraz gonadalne (to znaczy wywodzące się z komórek sznurów płciowych). Raki jajnika, jako nowotwory nabłonkowe, można podzielić w zależności od typu komórek, z których się wywodzą, na główne histotypy: surowicze, endometrioidalne, śluzowe, jasnokomórkowe, niezróżnicowane, mieszane oraz wywodzące się z nabłonka przejściowego (rzadkie złośliwe guzy Brennera) [5]. Już przed 10 laty Dubeau zauważył, że komórki te morfologicznie związane są z nabłonkiem jajowodu, endometrium, pęcherza moczowego lub przewodu pokarmowego, co oznacza, że wywodzą się z komórek, które w prawidłowych warunkach w jajniku nie występują [6]. Założył on, iż komórki te, wraz z komórkami nabłonkowymi jajnika mają wspólne pochodzenie z tkanek o budowie typu przewodu okołośródnerczowego Müllera. Komórki te zlokalizowane są w formie drobnych torbieli w okolicach jajników i jajowodów. Podobnie badania materiału zebranego od pacjentek będących nosicielkami mutacji *BRCA1 \ BRCA2*, u których została wykonana operacja profilaktycznego usunięcia jajników i jajowodów, wykazały, że głównym miejscem w którym odnajdywano zmienione dysplastycznie komórki nabłonka były dystalne (jajnikowe) końce jajowodów [7]. Co istotne, w żadnym z przebadanych preparatów nie znaleziono komórek nieprawidłowych w obrębie nabłonka jajników.

Kurman i wsp. po przeanalizowaniu dotychczasowych badań na temat pochodzenia raków jajnika zaproponowali ich podział na dwa typy (Tabela I) [8, 9]. Typ I stanowią guzy rosnące powoli na podłożu zmian łagodnych lub granicznie złośliwych (ang. *borderline tumors*) i stosunkowo późno dające przerzuty. Związane są z mutacjami genów *KRAS*, *BRAF*, *ERBB2*, *PTEN*, *PIK3CA* oraz *ARID1A*, a rzadko *TP53*. Cechują się lepszym rokowaniem niż raki typu II. Do tego typu należą raki o niskim stopniu złośliwości (ang. *low-grade*) surowicze, endometrioidalne, śluzowe oraz jasnokomórkowe. Natomiast do typu II zaliczane są agresywne, szybko rosnące i wcześnie dające przerzuty guzy o niższym zróżnicowaniu (ang. *high-grade*): surowicze, endometrioidalne, złośliwe mieszane guzy mezodermalne oraz guzy niezróżnicowane. Na poziomie molekularnym można stwierdzić mutacje *TP53* w ponad 80% przypadków, indeks proliferacji Ki67 zawiera się w przedziale 50-75%, a nadekspresję *HER2 Her2/neu* stwierdza się w 20-67% przypadków. Uważa się też, że od 10 do 15% raków jajnika jest dziedziczonych. Dotyczy to przede wszystkim zespołu dziedzicznego raka jajnika, w którym mutacje *BRCA1/2* stwierdza się w 90% przypadków. Do tego typu należą przede wszystkim raki surowicze o wysokim stopniu złośliwości (ang. *high-grade serous carcinoma*, *HGSC*), które stanowią około 70% wszystkich raków jajnika [8].

**Tabela 1:** Typy I i II nowotworów jajnika, na podstawie prac zespołu Kurmana [8, 9]. Table I. Types I and II of ovarian cancer, as based on works by the team of Kurman [8, 9].

	Domniemany prekursor *Putative precursor*	Najczęstsze mutacje genów *Most frequent genetic mutations present*	Stopień strukturalnych zaburzeń chromosomów *Level of chromosomal structural abberations*
**Typ I/*Type I***			
**Rak surowiczy o niskim stopniu złośliwości *Low-grade serous carcinoma***	surowiczy guz granicznie złośliwy *serous BOT*	*KRAS*, *BRAF*	niski *low*
**Rak endometrioidalny o niskim stopniu złośliwości *Low-grade endometrioid carcinoma***	endometrioza *endometriosis*	*CTNNB1*, *PTEN, ARID1A*	niski *low*
**Rak jasnokomórkowy *Clear cell carcinoma***	endometrioza *endometriosis*	*PIK3CA*, *ARID1A, FBXW74*	niski *low*
**Rak śluzowy *Mucinous carcinoma***	śluzowy guz granicznie złośliwy *mucinous BOT*	*KRAS*	niski *low*
**Typ II *Type II***			
**Rak surowiczy o wysokim stopniu złośliwości *High-grade serous carcinoma***	nabłonek jajowodu *tubal epithelium*	*TP53, BRCA1/2*	wysoki *high*
**Rak endometrioidalny o wysokim stopniu złośliwości *High-grade endometrioid carcinoma***	nieznany *unknown*	*TP53*	wysoki *high*
**Mięsakorak *Carcinosarcoma***	nieznany *unknown*	*TP53*	nieznany *unknown*

BOT - borderline ovarian tumor

Zgodnie z hipotezami dotyczącymi etiopatogenezy uważa się, że raki jajnika typu II pochodzą z komórek nabłonkowych jajowodu [9]. Najbardziej prawdopodobnym miejscem rozwoju *HGSC* jajnika jest dystalny odcinek jajowodu, z którego komórki następnie migrują do jajników [10]. Teoria ta opiera się głównie na dwóch obserwacjach. Pierwszą z nich są wyniki badań genetycznych wskazujące na podobną ilościowo, wysoką częstość mutacji *TP53* w zmianach przednowotworowych zlokalizowanych w jajowodach (ang. *serous tubal intraepithelial carcinoma*, *STIC*) oraz w rakach typu II. Drugą przesłankę stanowią wyniki analiz wielogenowych wskazujących na statystycznie znamienne i większe podobieństwo w ekspresji genów między rakami typu II, głównie *HGSC*, a komórkami jajowodów niż komórkami nabłonkowymi powierzchni jajnika. Dla przykładu, raki surowicze o wysokim stopniu złośliwości wykazują ekspresję białka PAX8 będącego markerem pochodzenia „Müllerowskiego”, a nie wykazują ekspresji kalretyniny będącej markerem nabłonka jamy otrzewnowej. Dodatkowo w pracy Singh i Cho sugeruje się, aby patomorfolodzy zwracali szczególną uwagę na możliwość, że wykryte zmiany nowotworowe jajników mogą być przerzutami z innych części żeńskiego układu rozrodczego [11]. Słuszności takiego podejścia dowodzi chociażby analiza porównawcza przypadków raków jajowodu w latach 1990-2002 oraz 2002-2012 wykonana w oparciu o dane North American Association of Central Cancer Registries [12]. Większa świadomość zagadnienia i ostrożność diagnostyczna patomorfologów dotycząca możliwości obecności komórek nowotworowych jako przerzutów raka jajowodu do jajnika już spowodowała znaczny wzrost wykrywania przypadków pierwotnego raka jajowodu. Właśnie biorąc pod uwagę powyższe teorie i dostępne dane naukowe zaproponowano możliwość pierwotnej profilaktyki raka jajnika przez usunięcie jajowodów. Dotychczas brakuje jednak twardych długoterminowych danych pozwalających na jednoznaczną ocenę skuteczności profilaktycznej obustronnej salpingektomii. Dlatego ważne jest rozważenie korzyści i ryzyka związanego z takim postępowaniem.

## Aktualne dane kliniczne

Średnioterminowe dane włoskie wskazują na bezpieczeństwo obustronnej salpingektomii dla utrzymania czynności pozostawionych jajników [13]. Również badania krótkoterminowe przynoszą zachęcające informacje, jednak różnią się stopniem efektywności. W 2010 r. kanadyjski British Columbia Ovarian Cancer Research Team, znany jako OVCARE, rozpoczął wdrażanie programu profilaktycznej salpingektomii szacując, że takie postępowanie zmniejszy ryzyko wystąpienia raka jajnika o połowę w ciągu najbliższych 20 lat. Wśród badań, na których oparto to założenie, znajduje się analiza przeprowadzona przez McAlpine i wsp. u ponad 43 000 pacjentek z grupy niskiego ryzyka, które poddały się obustronnej salpingektomii podczas histerektomii drogą laparotomii. Nie stwierdzono istotnych różnic w częstości wybranych powikłań śród- i pooperacyjnych, takich jak konieczność przetaczania krwi, ponowne hospitalizacje lub długość pobytu w szpitalu. Czas wykonania histerektomii z obustronną salpingektomią był średnio dłuższy jedynie o 16 minut w porównaniu do samej histerektomii [14]. Również inne badania dotyczące histerektomii metodą laparoskopową potwierdziły, że dodatkowa procedura nie wydłuża w sposób istotny czasu operacji oraz nie wydłuża ani pobytu w szpitalu, ani czasu powrotu do normalnej aktywności od chwili wypisania ze szpitala [15, 16, 17].

Obustronna profilaktyczna salpingektomia wydaje się być zabiegiem bezpieczniejszym w porównaniu do jednoczesnego usunięcia jajników i jajowodów. Pozostawienie jajników zabezpiecza przed wywołaniem jatrogennej menopauzy, dzięki czemu nie zwiększa ryzyka wystąpienia zdarzeń sercowo-naczyniowych, osteoporozy czy zaburzeń kognitywnych. W badaniach Morelli’ego i wsp. również nie stwierdzono istotnego wpływu na czynność jajników pomimo usunięcia jajowodów [13, 15]. M.in. nie stwierdzono zmian stężeń obwodowych hormonu anty-Müllerowskiego i folikulostymuliny, ani liczby pęcherzyków antralnych. Nie zmniejszyła się również średnica jajników. Z kolei badania dotyczące salpingektomii przeprowadzone przez Lin i wsp. [18] wskazały, że jajniki zachowały zdolność do prawidłowej odpowiedzi na stymulację gonadotropinami pomimo obustronnej salpingektomii, co pozwala na przeprowadzenie zapłodnienia pozaustrojowego, jeżeli pacjentka będzie chciała ponownie zajść w ciążę.

Przeprowadzono też analizę jakości życia oraz funkcji seksualnych u pacjentek, które przebyły różne typy histerektomii. Konkretnie, porównano oceny jakości życia oraz funkcji seksualnych u kobiet po usunięciu macicy drogą laparoskopową, brzuszną oraz przezpochwową, dodatkowo oceniając wpływ profilaktycznego usunięcia jajowodów [19]. W badaniu tym nie stwierdzono istotnej statystycznie różnicy pod względem jakości życia oraz funkcji seksualnych pomiędzy pacjentkami, u których przeprowadzono profilaktyczną salpingektomię, a tymi, u których jajowody zachowano.

Ważnym argumentem przemawiającym za wykonywaniem profilaktycznej obustronnej salpingektomii jest analiza stosunku kosztów do efektów przeprowadzona przez Diley i wsp. [20]. Stworzyli oni modele zestawiające koszty całego procesu diagnostycznego i leczniczego, w porównaniu do ewentualnych oszczędności uzyskanych dzięki usunięciu jajowodów w ramach profilaktyki pierwotnej. Dogłębna analiza problemu potwierdziła, że profilaktyczna salpingektomia wykonywana podczas histerektomii jest nie tylko zabiegiem korzystnym dla zdrowia pacjentek, ale też opłacalnym i umożliwiającym generowanie oszczędności w systemie ochrony zdrowia, wynikających z odjęcia kosztów ewentualnego leczenia rozwiniętych postaci nowotworów.

## Pacjentki będące nosicielkami mutacji *BRCA1 \ BRCA2*

Szczególne miejsce w rozważaniach dotyczących sensu przeprowadzenia profilaktycznego usunięcia jajowodów wraz z jajnikami zajmują pacjentki będące nosicielkami mutacji *BRCA1 \ BRCA2*. Zespół badaczy pod kierownictwem Finch przeprowadził obserwację pacjentek obciążonych genetycznie, porównując te, które przeszły zabieg profilaktycznego usunięcia przydatków (ang. *risk reducing salpingo-oophorectomy*, RRSO) oraz te, u których przydatki pozostawiono [21]. Mediana obserwacji wynosiła 3,5 roku. W ogólnej grupie 1828 pacjentek, wśród pacjentek z zachowanymi przydatkami wykryto 32 nowotwory: 29 raków jajnika, 2 raki jajowodu i 1 pierwotny rak otrzewnej. Wśród 490 kobiet, u których wykonano zabieg RRSO, u 11 z nich w preparatach pooperacyjnych wykryto komórki nowotworowe. Zestawiając powyższe dane z danymi populacyjnymi w USA, autorzy stwierdzili, że profilaktyczny zabieg RRSO u pacjentek będących nosicielkami mutacji *BRCA1* oraz *BRCA2* obniża ryzyko wystąpienia nowotworów jajnika, jajowodu lub pierwotnego raka otrzewnej aż o 80%.

W jednym z większych badań prospektywnych, przeprowadzonych przez Domchek i wsp. u 2482 pacjentek będących nosicielkami mutacji *BRCA1/2*, oszacowano stopień obniżenia ryzyka wystąpienia nowotworu oraz śmiertelności uwzględniając typ mutacji [22]. U kobiet będących nosicielkami mutacji *BRCA1*, u których wykonano RRSO, obniżenie ryzyka nowotworu jajnika oszacowano na poziomie 70-85%, natomiast u pacjentek z mutacją *BRCA2* - na poziomie 64%. Ponadto RRSO obniżało śmiertelność ogólną o 76%, natomiast tę związaną konkretnie z nowotworem piersi o 90%, a z nowotworem jajnika o 95%.

W Niemczech rocznie wykonywanych jest około 125 000 histerektomii. Według analizy zespołu Kommission Ovar, gdyby podczas wszystkich tych operacji usunięto profilaktycznie jajowody i gdyby spowodowałoby to wykrycie wszystkich nowych przypadków raka surowiczego jajnika, który wśród ~8000 nowych zachorowań stanowi 70%, to w efekcie w ciągu 20 lat nastąpiłoby zmniejszenie częstości występowania wszystkich raków jajnika do poziomu 2,3%. Jednak dla dokładniejszych szacunków konieczna jest ściślejsza obserwacja celem wykluczenia bądź odkrycia innych mechanizmów powstawania nowotworów jajnika [23].

O ile w przypadku profilaktycznego usunięcia jajowodów nie ma wątpliwości odnośnie braku ewentualnych konsekwencji dotyczących jakości życia w przyszłości, o tyle w przypadku usunięcia jajników konieczne było uwzględnienie ewentualnych następstw związanych z gospodarką hormonalną. W badaniach krótkoterminowych ocena jakości życia pacjentek po przebytym RRSO jest porównywalna z populacją ogólną oraz z tymi pacjentkami, u których stwierdzono nosicielstwo mutacji, ale pozostawiono je do wnikliwej obserwacji. Odczuwane są natomiast objawy związane z obniżeniem stężeń hormonów płciowych po usunięciu jajników. Pacjentki zgłaszały uderzenia gorąca, nocne poty oraz pogorszenie jakości życia seksualnego związane z suchością pochwy oraz bolesnymi stosunkami. Jednak mimo to pacjentki, które poddały się RRSO, stwierdzały wysoki poziom zadowolenia z podjętej decyzji oraz były mniej obciążone obawami związanymi z rozwojem choroby nowotworowej w stosunku do nosicielek mutacji *BRCA1*, które nie miały wykonanego RRSO, a pozostawały jedynie w obserwacji. Wciąż brakuje natomiast danych dotyczących wpływu na jakość życia i zdrowia pacjentek w obserwacjach długoterminowych [24, 25].

## Ocena salpingektomii przez międzynarodowe towarzystwa naukowe i grupy eksperckie

Najnowsze wytyczne amerykańskiej National Comprehensive Cancer Network (NCCN), tj. sieci 26 wiodących ośrodków onkologicznych w USA, dotyczące postępowania profilaktycznego oraz diagnostyczno-leczniczego w raku jajnika wskazują na zasadność rozważenia profilaktycznej salpingektomii. Zaznaczają jednak, że rola i wartość takiej procedury w profilaktyce raka jajnika nie jest jeszcze jednoznacznie udowodniona i należy pamiętać o możliwym wystąpieniu takiego nowotworu po salpingektomii, choć ryzyko to jest mniejsze, ale trudne do oszacowania. Ponadto, autorzy zawracają uwagę na wpływ usunięcia jajników na zmniejszenie ryzyka raka piersi, zwłaszcza u kobiet w okresie przedmenopauzalnym, choć siła tego wpływu również nie jest jeszcze jednoznacznie określona [26]. Z kolei najnowsze wytyczne tej samej organizacji dotyczące obniżenia ryzyka raka piersi i jajnika u kobiet obarczonych mutacjami w genach *BRCA* są bardziej restrykcyjne i podają, że salpingektomia nie jest standardem profilaktyki raka jajnika, choć zwracają uwagę na toczące się badania oceniające rolę i bezpieczeństwo salpingektomii z odroczonym usunięciem jajników (ang. *interval salpingectomy with delayed oophorectomy*) [27].

Z drugiej strony najnowsze wytyczne European Society for Medical Oncology (ESMO) mówiące o roli RRSO w zmniejszaniu ryzyka raka piersi u nosicielek mutacji BRCA1/2 kierują uwagę na ostatnie doniesienia z prospektywnego badania kohortowego, w którym, wbrew wcześniejszym doniesieniom z licznych badań (głównie retrospektywnych), nie wykazano zmniejszenia ryzyka raka piesi u kobiet poddanych RRSO. Stąd eksperci ESMO traktują profilaktyczną salpingektomię jako wciąż metodę eksperymentalną i możliwą do stosowania wyłącznie w ramach badań klinicznych [3].

Zdaniem przedstawicieli American College of Obstetricians and Gynecologists (ACOG) salpingektomia może stanowić metodę zapobiegania rakowi jajnika z zastrzeżeniami, iż: 1) dotyczy to kobiet o populacyjnym ryzyku raka jajnika (z wyłączeniem zatem pacjentek o wysokim ryzyku, u których zidentyfikowano podłoże genetyczne zwiększające ryzyko tych nowotworów), 2) dotyczy to kobiet poddawanych histerektomii z powodu innych chorób oraz, że 3) procedura ta wciąż wymaga kontrolowanych badań randomizowanych mających na celu ocenę redukcji ryzyka nowotworzenia oraz ocenę możliwości występowania nowotworów w pozostawionych jajnikach [4].

Co ciekawe, tegoroczna praca przeglądowa autorstwa znanych kanadyjskich onkologów [28] posuwa się jeszcze dalej i sugeruje istotną rolę usuwania przydatków zajętych endometriozą w profilaktyce raków jajnika mogących wywodzić się z endometriozy (Tabela I).

## Podsumowanie

Reasumując, analiza powyższych aspektów i przytoczonych badań przemawia za licznymi korzyściami z wykonywania profilaktycznej salpingektomii. Biorąc pod uwagę dostępne dane literaturowe zabieg ten może w znaczący sposób obniżyć zachorowalność na raka jajnika. Jednocześnie jest on bezpieczny, nie wpływa w sposób istotny na funkcjonowanie jajników po operacji, nie pogarsza jakości życia ani funkcjonowania seksualnego pacjentek, nie wydłuża znacząco czasu zabiegu operacyjnego oraz nie powoduje zwiększonej utraty krwi podczas operacji. Zaprezentowana analiza kosztów dowodzi, że profilaktyczna salpingektomia może generować znaczące oszczędności w systemie opieki zdrowotnej. Niewątpliwie brakuje długoterminowych badań oceniających odległe efekty takiego postępowania, szczególnie w odniesieniu do redukcji występowania nowotworów jajnika. Uzasadnia to przeprowadzenie takich badań na dużych grupach pacjentek, a ich wyniki ostatecznie pomogą rozstrzygnąć czy profilaktyczna obustronna salpingektomia stanie się powszechnie przyjętym elementem profilaktyki pierwotnej raka jajnika, przynajmniej w populacji kobiet nieobciążonych genetycznie.

## References

[1] Didkowska J, Wojciechowska U (2015). Nowotwory złośliwe w Polsce w 2013roku. Krajowy Rejestr Nowotworów.

[2] Markowska A (2015). Epidemiologia i etiopatogeneza raka jajnika W: Markowska J, Mądry R (Red.) Zarys Ginekologii Onkologicznej. Tom II.

[3] Paluch-Shimon S, Cardoso F, Sessa C, Balmana J, Cardoso MJ, Gilbert F, Senkus E (2016). on behalf of the ESMO Guidelines Committee. Prevention and screening in BRCA mutation carriers and other breast/ovarian hereditary cancer syndromes: ESMO Clinical Practice Guidelines for cancer prevention and screening. Ann Oncol.

[4] (2015). Committee Opinion No. 620. Salpingectomy for ovarian cancer prevention. Obstet Gynecol.

[5] WHO Classification of Ovarian Neoplasms. PathologyOutlines. com.

[6] Dubeau L (2008). The cell of origin of ovarian epithelial tumours. Lancet Oncol.

[7] Tone AA, Salvador S, Finlayson SJ, Tinker AV, Kwon JS, Lee CH, Cohen T, Ehlen T, Lee M, Carey MS (2012). The role of the fallopian tube in ovarian cancer. Clin Adv Hematol Oncol.

[8] Kurman R, Shih I-M (2010). The origin and pathogenesis of epithelial ovarian cancer – a proposed unifying theory. Am J Surg Pathol.

[9] Nik NN, Vang R, Shih I-M, Kurman RJ (2014). Origin and pathogenesis of pelvic (ovarian, tubal, and primary peritoneal) serous carcinoma. Annu Rev Pathol Mech Dis.

[10] Kindelberger DW, Lee Y, Miron A, Hirsch MS, Feltmate C, Medeiros F, Callahan MJ, Garner EO, Gordon RW, Birch C (2007). Intraepithelial carcinoma of the fimbria and pelvic serous carcinoma: evidence for casual relationship. Am J Surg Pathol.

[11] Singh R, Cho KR (2017). Serous tubal intraepithelial carcinoma or not? Metastases to fallopian tube mucosa can masquerade as in situ lesions. Arch Pathol Lab Med.

[12] Trabert B, Coburn SB, Mariani A, Yang HP, Rosenberg PS, Gierach GL, Wentzensen N, Cronin KA, Sherman ME (2018). Reported incidence and survival of fallopian tube carcinomas: a population-based analysis from the North American Association of Central Cancer Registries. J Natl Cancer Inst.

[13] Venturella R, Lico D, Borelli M, Imbrogno MG, Cevenini G, Zupi E, Zullo F, Morelli M (2017). 3 to 5 years later: Long-term effects of prophylactic bilateral salpingectomy on ovarian function. J Minim Invasive Gynecol.

[14] McAlpine JN, Hanley GE, Woo MMM, Tone AA, Rozenberg N, Swenerton KD, Gilks CB, Finlayson SJ, Huntsman DG, Miller DM for the Ovarian Cancer Research Program of British Columbia. (2014). Opportunistic salpingectomy: uptake, risks, and complications of a regional initiative for ovarian cancer prevention. Am J Obstet Gynecol.

[15] Morelli M, Venturella R, Mocciaro R, Di Cello A, Rania E, Lico D, D’Alessandro P, Zullo F. (2013). Prophylactic salpingectomy in premenopausal low-risk women for ovarian cancer: Primum non nocere. Gynecol Oncol.

[16] Kwon JS (2015). Ovarian cancer risk reduction through opportunistic salpingectomy. J Gynecol Oncol.

[17] Finlayson S for the BC’s Ovarian Cancer Research Team. Advocating Fallopian Tube removal at the time of hysterectomy to prevent ovarian cancer.

[18] Lin YJ, Ou YC, Huang FJ, Lin PY, Kung FT, Lan KC (2013). Ovarian response to gonadotropins in patients with tubal factor infertility: salpingectomy versus nonsalpingectomy. J Minim Invasive Gynecol.

[19] Skorupska KA, Miotła P (2016). Are there any differences in quality of life and sexual functions after various types of hysterectomy – does prophylactic salpingectomy matter?. Ginekol Pol.

[20] Dilley SE, Havrilesky LJ, Bakkum-Gamez J, Cohn DE, Straughn Jr JM, Caughey AB, Rodriguez MI. (2017). Cost-effectiveness of opportunistic salpingectomy for ovarian cancer prevention. Gynecol Oncol.

[21] Finch A, Beiner M, Lubinski J, Lynch HT, Moller P, Rosen B, Murphy J, Ghadirian P, Friedman E, Foulkes WD (2006). for the Hereditary Ovarian Cancer Clinical Study Group. Salpingo-oophorectomy and the risk of ovarian, fallopian tube and peritoneal cancers in women with a BRCA1 or BRCA2 mutation. JAMA.

[22] Domchek SM, Friebel TM, Singer CF, Evans DG, Lynch HT, Isaacs C, Garber JE, Neuhausen SL, Matloff E, Eeles R (2010). Association of risk-reducing surgery in BRCA1 or BRCA2 mutation carriers with cancer risk and mortality. JAMA.

[23] Pölcher M, Hauptmann S, Fotopoulou C, Schmalfeldt B, Meinhold-Heerlein I, Mustea A, Runnebaum I, Sehouli J (2015). for the Kommission Ovar of the Gynecologic Oncology Study Group (AGO). Should fallopian tubes be removed during hysterectomy procedures? – A statement by AGO Ovar / Sollen die Tuben im Rahmen der Hysterektomie entfernt werden? – Ein Statement der AGO Ovar. Geburtshilfe Frauenheilkd.

[24] Finch A, Narod SA (2011). Quality of life and health status after prophylactic salpingo-oophorectomy in women who carry a BRCA mutation: a review. Maturitas.

[25] Finch A, Metcalfe KA, Chiang JK, Elit L, McLaughlin J, Springate C, Demsky R, Murphy J, Rosen B, Narod SA (2011). The impact of prophylactic salpingo-oophorectomy on menopausal symptoms and sexual function in women who carry a BRCA mutation. Gynecol Oncol.

[26] National Comprehensive Cancer Network.

[27] National Comprehensive Cancer Network.

[28] Dawson A, Fernandez ML, Anglesio M, Yong PJ, Carey MS (2018). Endometriosis and endometriosis-associated cancers: new insights into the molecular mechanisms of ovarian cancer development. Ecancermedicalscience.

